# Caregiver strain among patients of palliative care in Sri Lanka: validation of modified caregiver strain index - Sinhala version

**DOI:** 10.1186/s12904-023-01270-w

**Published:** 2023-11-04

**Authors:** U. Ramadasa, S. Silva, U. Udumulla, S. Perera, S. Lekamwasam

**Affiliations:** 1 Faculty of Medicine, Sabaragamuwa University of Sri Lanka, Ratnapura, Sri Lanka; 2 Faculty of Medical Sciences, University of Sri Jayewardenepura, Nugegoda, Sri Lanka; 3 Ministry of Health, Nutrition and Indigenous Medicine, Colombo, Sri Lanka; 4 Faculty of Medicine, University of Ruhuna, Galle, Sri Lanka

**Keywords:** Palliative Care, Caregiver strain, Caregiver Burden, Caregiver strain screening tools, Caregiver Burden Screening Tool, Modified caregiver strain index

## Abstract

**Background:**

Care givers of Palliated patients are at risk of adverse physical, psychosocial and emotional sequelae in varied nature. Efficient and valid assessment tools facilitate early detection to take corrective measures. The Modified Caregiver Strain Index (MCSI), composed of domains associated with caregiver strain is a simple and brief tool that can be used in both clinical and field settings. This study aimed to adapt and validate this in order to cater effective palliative care services in Sri Lanka.

**Methods:**

After cross-cultural adaptation, 200 primary caregivers in 3 teaching hospitals were recruited. The internal consistency, item-total correlations, of the 13-item S-MCSI were performed. The criterion validity was assessed by Pearson correlation between the total scores of S-MCSI, the Karnofky Performance Scale and the Barthel index. Construct validity was determined by the principal component analysis keeping the Varimax with Keiser normalization as the rotation method. The Kaiser-Meyer-Olkin test (KMO) and Bartlett’s test of sphericity statistics were also performed to determine the adequacy of the sample and correlations between items, respectively. The number of factors was determined by the Scree plot, percentage of variance explained by each component and number of Eigen values over 01 (Kaiser-Guttman rule).

**Results:**

The total MCSI score ranged 0 to 26. The overall Cronbach’s alpha of the 13-item questionnaire was 0.80 while item-total corrections ranged 0.34 to 0.62, exception of one item (0.11). Inverse correlations were demonstrated in total scores of MCSI and Karnofky Performance Scale (r =- 0.32, p < 0.001) and Barthel index (r =-0.34, P < 0.001). A Kaiser-Meyer-Olkin value of 0.79 (p < 0.001) for Bartlett’s test indicated adequate sampling and nonlinearity of factors. The Scree plot showed a three-factor structure explaining 57% of the variation. Items regarding personal wellbeing of caregiver loaded together while the effects on the family loaded separately. Adjustment of personal concerns and family issues along with time alteration grouped as the third factor.

**Conclusions:**

The study showed that the Sinhala version of MCSI has adequate psychometric properties and reliability to be used as a validated tool to estimate the caregiver burden within a short time period for any health care workers.

## Introduction

Palliative care is an organized interdisciplinary care approach intended to enhance the quality of life of patients with life-threatening illnesses as well as their families [1]. A family caregiver or informal caregiver refers to an unpaid family member, friend, or neighbour (other than health care providers) who provides care to an individual. The cared individual may have an acute or chronic condition needing assistance to manage a variety of tasks such as physical activities of daily living, administering medications, tube feeding, home oxygen delivery etc. [2] In addition to the patients, palliative care plans should incorporate informal caregivers since a terminal or incurable illness can potentially affect other members of the family. It is well-documented that family caregivers are prone to adverse physical, psychological, social and emotional sequelae [3]. These can potentially affect their well-being and quality of life. Hence, palliative care plans should be holistic, individualised and family-centred [4]. This is required as families with such patients need to make various adjustments and sacrifices to cope with the increased workload and stressors. Regular communication with family caregivers, especially information provision, is shown to lessen the burden of the caregiver. This is an important aspect of end-of-life care [5].

In Sri Lanka, there is a deficiency of pragmatic care guidance with suboptimal information delivery. This is due to poor awareness, lack of cooperation from healthcare workers and restricted provision of technical equipment for caregivers of palliated patients. The enhancement of awareness and improvement of non-technical skills of health care professionals are likely to be beneficial in the alleviation and prevention of caregiver burden [6]. Recent studies have highlighted the need of estimating the impact of caring on caregivers to recognize the additional burden cast upon them [7]. This will assist in reducing their burden through early recognition and timely interventions [8].

McDonald et al., in an interventional study, found that caregiver quality of life is greatly determined by the interaction with the patient and the authors recommended measuring the quality of life of caregivers with specific questionnaires that include content related to confronting mortality and professional supports [9].

The palliative care needs of cancer patients and their family caregivers are not adequately studied in Sri Lanka. A previous study by Ramadasa, et al. in a suburb of Sri Lanka has highlighted the hardships faced by family caregivers including financial difficulties, loss of income, transport difficulties, inability to engage in routine activities, and negative impact on job and education [10]. It was also revealed that the domiciliary caregivers of cancer patients have inadequate knowledge, experience and skills to provide palliative care. Malignancy is the second commonest cause of hospital mortality in Sri Lanka and epidemiological data have shown an upward trend in the incidence of malignancies in the country. Furthermore, the overall incidence of cancer has doubled over the past 25 years in Sri Lanka [11]. This will lead to a considerable burden on health care systems, supportive services and families of those affected. Hence family-based palliative care programs should be expanded to meet the increased demand.

The current palliative care programs in Sri Lanka do not incorporate an assessment of caregiver burden. This gap can be attributed to both poor awareness and the lack of validated and reliable tools to estimate the caregiver burden in the local community. The Modified Caregiver Strain Index (MCSI) represents a set of domains associated with caregiver strain. It is composed of 13 questions which are answered as 3 response choices: never (0 marks), sometimes (1 mark), always (2 marks). The cumulative score is calculated, thus with a range of 0 to 26. Higher scores reflect higher levels of burden. No defined cut-off was determined by the investigators for MCSI. However, a cumulative score of 7 or more is considered as significant [12]. This is a simple tool developed by Thornton and Travis (2003) with a limited number of questions that can be used in the community as well as in busy clinical settings [13]. It is therefore advantageous to be used in overcrowded and restricted outpatient and inpatient setting as in the health sector of Sri Lanka. The original Caregiver Strain Index (CSI) tool had a two step answer system which deterred the responses of the interviewee. A middle level response introduced in the MCSI has averted this allowing the respondents to provide a appropriate choice when needed. It is also shown that by careful consideration of varying nature of ethnic backgrounds and health-socioeconomic inequalities will assist in tailor made interventions. The cons of usage of this tool in South Asian context is, that it was originally developed in the setting of a Western Culture. There is no cultural adaptation found in the Indian Subcontinent, therefore requiring a necessity in attempting an adaptation. Furthermore the MCSI tool does not have ordinal categorisation of scores as an accepted means. A keen professional insight is needed to interpret the total score of the caregiver burden [14].

The MCSI has been culturally adapted and validated in many countries to estimate caregiver strain in numerous conditions [15].– [16].

## Methodology

Consenting primary caregivers of palliated cancer patients from three cancer care units in Sri Lanka; Apeksha Hospital at Maharagama and Teaching Hospitals at Rathnapura and Colombo South were recruited for the study. Assuming 80% power of the study, Cronbach’s alpha for the null hypothesis of 0.5 and expected Cronbach’s alpha of 0.7, a sample of 190 was required. We recruited 200 primary caregivers of patients who presented to the study settings by purposive sampling method.

In selecting caregivers, patients with advanced malignancies directed for palliative care were considered regardless of the type and site of malignancy. Patients who were in remission or were being treated with curative intent were excluded. Caregivers were interviewed with the translated MCSI questionnaire as well as other in depth questions pertaining to socioeconomic status and patients medical and functional domains. This was done in absence of patients and other members of the family in a place where privacy could be maintained. Data were collected by medical officers who were informed about the study and trained to collect the necessary information.

The cross-cultural adaptation of the MCSI was performed adhering to the standard guidelines described by Beaton et al. [17]. The original English version was translated into the Sinhala language by two independent health professionals conversant in both languages. One person was informed of the details of the study while the other was not. The two translations were consolidated into one document by the principal investigator in the presence of the two translators. This was done to improve the clarity, ease of comprehension and unambiguity of the items. This version was reverse-translated to English by two different health professionals to determine the comparability with the original version. A group of experts consisting of two physicians, one oncologist and one community physician together with the principal investigator reviewed the translated questionnaire to ensure clarity, face validity, content validity and semantic equivalence. Thus the English version was culturally adapted item by item for the Sinhala language.

The pre-final version was piloted among 30 caregivers selected from two study centres to ensure the internal consistency of the questionnaire. The final version (S-MCSI) was administered to 192 consented caregivers of patients with incurable malignancies, undergoing palliative care in study settings. Informed written consent was obtained from the participants before data collection. The tool was an interviewer-administered questionnaire without prompting. The Karnofky Performance Scale and the Sinhala-translated version of the Barthel index were also used in data collection [18].

The internal consistency of the 13-item S-MCSI was examined with Cronbach’s alpha and item-total correlations. The criterion validity was assessed by Pearson correlation between the total scores of S-MCSI, the Karnofky Performance Scale and the Barthel index. Construct validity was determined by the principal component analysis keeping the Varimax with Keiser normalization as the rotation method. The Kaiser-Meyer-Olkin test (KMO) and Bartlett’s test of sphericity statistics were also performed to determine the adequacy of the sample and correlations between items, respectively. The number of factors was determined by the Scree plot, the percentage of variance explained by each component and the number of Eigen values over 01 (Kaiser-Guttman rule). Items were considered representative of a component if their item loading was ≥ 0.40 and in the cross-loading items, the factor, which had a higher loading value, was considered to determine the positioning of the respective factor.

Ethical clearance for the study was obtained from the Ethics Review Committee of the Faculty of Medicine, Sabaragamuwa University of Sri Lanka. The study was performed adhered to the ethical standards stated in the Declaration of Helsinki [19].

## Results

A total of 200 caregivers (74% women) with a mean (standard deviation) age of 46.2 (15.1) years completed the S-MSCI. Sixty-eight per cent of the primary caregivers were children of the patients (mean age = 42.5 years, SD 18.1, Range 15–65) while 30% and 2% were spouses and other relatives, respectively. The mean age (standard deviation) of patients (64% women) undergoing palliative care was 62.0 (12.4) years. (Table 1)

**Table 1:** Characteristics of caregivers and their patients

**Ages**	
Mean Age (SD) of Caregivers in years	46.2 (15.1), Range 15–75
Mean Age (SD) of Patients	62.0 (12.4), Range 6–88
**Sex of Caregiver**	
Male	50 (26%)
Female	150 (74%)
**Relationship to Patient**	
Children	136 (68%)
Spouses	60 (30%)
Other relatives	4 (2%)
**Level of Education of Caregiver**	
Never schooled	2 (1%)
Primary education	32 (16%)
GCE Ordinary Level examination	68 (34%)
GCE Advanced Level examination	79 (39.5%)
Vocational training	11 (5.5%)
University level	8 (4%)
**Monthly Household Earnings (USD)**	
< 50	5 (2.5%)
50–100	15 (7.5%)
100–250	55 (27.5%)
250–500	124 (62%)
> 500	1 (0.5%)
**Type of Malignancy**	
Oral & Lingual	3 (1.5%)
Nasopharyngeal	3 (1.5%)
Laryngeal	2 (1%)
Broncheal	12 (6%)
Gastro-oesophageal	15 (7.5%)
Colorectal	40 (20%)
Hepatocellular	5 (2.5%)
Biliary	4 (2%)
Urological (including prostate & bladder)	15 (7.5%)
Haematological (leukaemia, lymphoma, multiple myeloma)	52 (26%)
Breast	18 (9%)
Gynaecological	20 (10%)
Penile and testicular	2 (1%)
Primary Cerebral	5 (2.5%)
Primary Bone	4 (2%)
**Modified Barthel Index Score of Patients**	
Total dependency (≤ 20)	12 (6%)
Severe dependency (21–60)	31 (15.5%)
Moderate dependency (61–90)	77 (38.5%)
Mild dependency (91–99)	76 (38%)
Independence (100)	4 (2%)
**Karnofsky Performance Status of Patients**	
No complaints; no evidence of disease (100%)	1 (0.5%)
Able to carry on normal activity; minor signs or symptoms of disease (90%)	34 (17%)
Normal activity with effort; some signs or symptoms of disease (80%)	72 (36%)
Cares for self; unable to carry on normal activity or to do active work (70%)	47 (23.5%)
Requires occasional assistance but is able to care for most personal needs (60%)	18 (9%)
Requires considerable assistance and frequent medical care (50%)	10 (5%)
Disabled; requires special care and assistance (40%)	5 (2.5%)
Severely disabled; hospital admission indicated although death not imminent (30%)	2 (1%)
Very sick; hospital admission necessary; active supportive treatment necessary (20%)	2 (1%)
Moribund; fatal processes progressing rapidly (10%)	1 (0.5%
Dead (0%)	0 (0%)

The total S-MCSI score ranged from 0 (three subjects) to 26 (two subjects). The overall Cronbach’s alpha of the 13-item questionnaire was 0.80 while the item-total corrections varied from 0.34 to 0.62, except for item 12 which showed a correlation of 0.11 (Table 2).

**Table 2:** Corrected item-total correlations of the 13 items in the S-MCSI

Item number	Item	Corrected item-total correlation	Cronbach’s alpha if the item is deleted
1 2 3 4 5 6 7 8 9 10 11 12 13	Sleep is disturbed Inconvenience Physical strain Confining Family adjustments Change in personal plans Other demands on time Emotional adjustments Upsetting behaviour Upsetting change of self Work adjustment Financial strain Complete overwhelmed	0.46 0.45 0.54 0.62 0.52 0.59 0.35 0.45 0.51 0.45 0.49 0.11 0.38	0.79 0.80 0.79 0.78 0.79 0.78 0.80 0.79 0.79 0.79 0.79 0.83 0.80

**Fig. 1:**
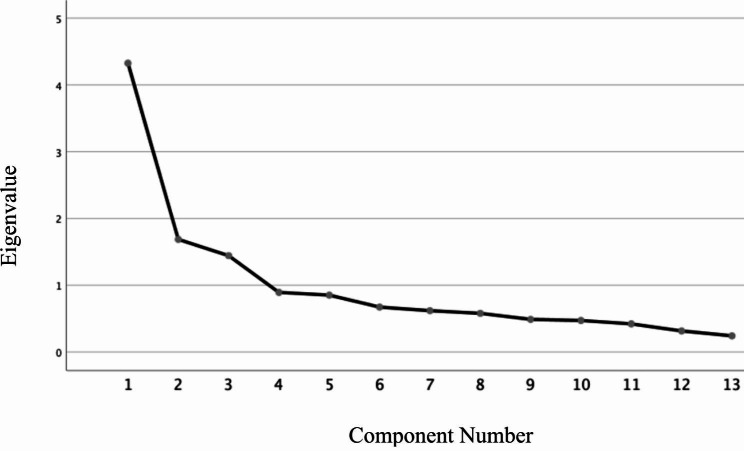
Scree plot with factors

In criterion validity analysis, inverse correlations were observed between the total scores of S-MCSI and Karnofky Performance Scale (r =- 0.32, p < 0.001) and Barthel index (r =-0.34, P < 0.001). Factor analysis showed a KMO value of 0.79 (p < 0.001) for Bartlett’s test indicating adequate sampling and nonlinearity of factors. The principal component analysis (Eigenvalue > 1) and the Scree plot (Fig. 1.) showed a three-factor structure explaining 57% of the variation. Items 1, 2, 3, 8, 9 and 10 loaded on factor 1, while items 4 to 7 loaded together on factor 2. Items 12 and 13 are loaded separately on factor 3 (Table 3).

**Table 3:** Rotated component matrix showing factor loading

Item number	Item	Factor 1	Factor 2	Factor 3
1 2 3 4 5 6 7 8 9 10 11 12 13	Sleep is disturbed Inconvenience Physical strain Confining Family adjustments Change in personal plans Other demands on time Emotional adjustments Upsetting behaviour Upsetting change of self Work adjustment Financial strain Complete overwhelmed	0.51 0.62 0.73 0.84 0.80 0.48	0.73 0.84 0.81 0.48	0.86 0.83

The Scree plot in determining the number of factors is shown below (Fig. 1).

## Discussion & conclusions

The high demand for care results in a huge burden. There is also stress generated due to empathetic suffering. There is a feeling of being overwhelmed and enduring financial and physical burdens [20]. The utilisation of caregiving within the family and how it is being trained have been studied. Caregiving in a home setting is an important component of patient care, while this experience is a noteworthy component that needs to be addressed to reduce the burden on the caregiver with the provision of appropriate support [21]. Studies by Schulz and Ugor have stated a multitude of care recipient-related determinants of caregiver strain including behaviour problems, functional disabilities and cognitive functions of the care recipient and the duration and intensity of care provided [3, 16]. Most of these factors are potentially modifiable or avoided if sufficient attention is paid.

Caregiver assessment tools are required to identify the individual needs that are poorly identified. An efficient and valid method permits early detection of these needs and strains to take remedial actions. Furthermore, screening tools are helpful to identify those that are at high risk [22].

Most studies conducted to assess or screen the burden of caregiving have used quantitative measures which are not so effective and valid in measuring the gravity of the problem. The attitudes and commitment of caregivers and the stresses experienced in the process are likely to vary between cultures due to the differences in values, beliefs and attitudes. A cultural adaptation is therefore much needed when translating a tool into a different language.

Many tools related to caregiving have been translated into different languages to suit local populations (need a couple of references). This is logical as the needs of care recipients and the strain on caregivers are likely to vary in different cultures and ethnicities. The S-MCSI will identify different stressors faced by caregivers who are conversant in Sinhala. Subsequently, this information can be used in designing palliative care plans in Sri Lanka.

Our analysis demonstrates that the S-MCSI has adequate psychometric properties to be used as a validated tool to estimate the caregiver burden. The short time required to fill in the information and the convenience of administration allows a range of health care professionals including doctors, nurses and even social workers to gather this information.

The systematic translation and validation process ensured that the S-MCSI has psychometric properties similar to the original MCSI. While the original English version had an overall Cronbach’s alpha of 0.90 [13], the Turkish [16] and Malaysian versions [23] showed marginally lesser internal consistencies of 0.77 and 0.79, respectively. These values are concordant with the internal consistency of 0.80 we observed. The factor analyses of S-MCSI have shown varying results and this could partly be due to the cultural and religious variations of study subjects. We observed a 3-factor structure of the questionnaire.

The item-total correlations seen in this analysis show a high level of measurement reliability. In the factor analysis, items regarding the personal well-being of the caregiver such as effects on sleep, inconvenience, physical and mental strain and emotional adjustment loaded together (factor 1) while the impact on the family showed different loading (factor 2). Furthermore, adjustment of personal concerns and family issues along with alteration of time demands loaded together as factor 3.

The Turkish validation study showed a 4-factor structure which the authors had described in terms of themes [16]. We observe that such similar themes emerge in our study as well. It is noteworthy that factor 1 of S-MCSI was comparable to combined factors 2 and 3 of the Turkish version which were themed as ‘upsetting’ and ‘inconvenient’. The items in factors 2 and 3 of the Sinhala version were similar to Turkish factors 1 and 4, described as ‘adaptation’ and ‘overwhelming’ respectively.

We observe that there is some reluctance of caregivers to discuss the financial strains involved with caregiving and this again can be due to cultural and religious factors. The main religions practised in Sri Lanka; Buddhism, Christianity, Islam and Hinduism highlight the importance of love and care, particularly for those who are sick. These values are inculcated very early in life and may determine the way caregivers handle the pressure and strain associated with the long-term caregiving of their loved ones [24, 25]. Furthermore, patients with malignancies, especially those who are incurable, receive the sympathy of people and it is not uncommon for caregivers of cancer patients to show more resilience.

We conclude that the S-MCSI is a valid tool to estimate the burden and stress among Sinhala-conversant caregivers of cancer patients in Sri Lanka. This will help in identifying the caregivers who are under stress and their need to provide professional assistance to mitigate. Furthermore, measures to reduce the burden on caregivers can be included in holistic family-centred palliative care plans.

The current study has a few limitations. The study sample included only the caregivers of cancer patients selected from three study locations. The study needs to be done at multiple centres in Sri Lanka representing all socio-economic and geographical variations. The applicability of this tool to caregivers of patients with other diseases such as end-stage kidney and liver disease needs to be established. Similarly, the questionnaire needs to be translated into Tamil to be validated as it is another national language of Sri Lanka. The questionnaire needs to capture various ethnic communities separately as each community may be speaking 1 or more official languages in Sri Lanka. Further qualitative studies should be done to identify and analyse culturally unique thoughts and emotions [20]. These are the possible future research oppurtuinities that are generated from the validation of MCSI-S.

The study will open dialogue for researchers to study the burden of caregiver strain in the context of palliated patients in Sri Lanka. Thus demographic, socio-economic and disease based patterns can be studied in consideration of the caregiver burden. Palliative care is a new field in Sri Lanka. The data generated regarding the strain of the care takers will be instrumental in designing and monitoring new strategies to develop this field.

## Data Availability

The datasets used and/or analysed during the current study available from the corresponding author on reasonable request.
